# Geroprotective effects of Alzheimer’s disease drug candidates

**DOI:** 10.18632/aging.202631

**Published:** 2021-02-06

**Authors:** Devin Kepchia, Antonio Currais, Richard Dargusch, Kim Finley, David Schubert, Pamela Maher

**Affiliations:** 1 Cellular Neurobiology Laboratory, The Salk Institute for Biological Studies, La Jolla, CA 92037, USA; 2 Donald P. Shiley BioScience Center, San Diego State University, San Diego, CA 92115, USA

**Keywords:** geroprotection, dementia, chronic kidney disease (CKD), comorbidity, inflammation

## Abstract

Geroprotectors are compounds that slow the biological aging process in model organisms and may therefore extend healthy lifespan in humans. It is hypothesized that they do so by preserving the more youthful function of multiple organ systems. However, this hypothesis has rarely been tested in any organisms besides *C. elegans* and *D. melanogaster*. To determine if two life-extending compounds for *Drosophila* maintain a more youthful phenotype in old mice, we asked if they had anti-aging effects in both the brain and kidney. We utilized rapidly aging senescence-accelerated SAMP8 mice to investigate age-associated protein level alterations in these organs. The test compounds were two cognition-enhancing Alzheimer’s disease drug candidates, J147 and CMS121. Mice were fed the compounds in the last quadrant of their lifespan, when they have cognitive deficits and are beginning to develop CKD. Both compounds improved physiological markers for brain and kidney function. However, these two organs had distinct, tissue-specific protein level alterations that occurred with age, but in both cases, drug treatments restored a more youthful level. These data show that geroprotective AD drug candidates J147 and CMS121 prevent age-associated disease in both brain and kidney, and that their apparent mode of action in each tissue is distinct.

## INTRODUCTION

As life expectancy increases worldwide, so too does the number of years spent living with a chronic illness. People over 60 years of age are currently 12 percent of the world’s population and this demographic is expected to double by the year 2050, reaching 2 billion people [1]. Without a parallel increase in healthspan, medical care for the increasingly elderly population will have enormous economic and social consequences [2].

Currently, no approved treatments are able to stop or slow the progression of Alzheimer’s disease (AD). The AD brain is pathologically characterized by amyloid plaques and neurofibrillary tangles, along with synaptic and neuronal loss [3]. It is likely that therapeutic progress has failed due to the pharmaceutical industry’s focus on preselected targets, such as those in the β-amyloid pathway [4–6]. Partly due to these failures, the pharmaceutical industry has shifted its interest towards tau-targeting therapies for AD. However, strategies that focus on inhibition of tau kinases or tau aggregation, or on stabilization of microtubules have mostly been discontinued due to toxicity and/or lack of efficacy. Currently, immunotherapies comprise the majority of tau-targeting therapies in clinical trials [3]. However, we recently assessed protein aggregation in the brains of AD patients and found that tau pathology in the context of its polymerization and insolubility was not associated with the majority of AD patients within our study’s cohort, suggesting caution when using tau as a universal drug target for AD [7].

Instead of working within these narrow limitations, we developed a multimodal drug screening approach that mimics the physiological changes that take place in the aging brain [8]. This paradigm includes four toxicity assays that assess cell survival under conditions of low energy metabolism, oxidative stress, proteotoxicity, and reduced trophic factor support, as well as an assay for anti-inflammatory activity. As most of these insults occur in multiple tissues throughout the body during aging, we hypothesize that favorably performing compounds will also be beneficial to other organs besides the brain that are impacted by age-related diseases.

Kidney disease is often another age-dependent ailment. Chronic kidney disease (CKD), a general term that describes a loss of structural and functional renal integrity, leads to a reduced glomerular filtration rate and a subsequent buildup of uremic toxins that negatively impact other organs throughout the body [9]. CKD is associated with aging, while diabetic kidney disease (DKD) develops in nearly 40% of type II diabetic patients and is a leading cause of CKD [10, 11]. A clinical correlation exists between patients with kidney disease and neurological dysfunction, including mild cognitive impairment (MCI) and dementia [12–14]. Forty-five percent of adults over 70 have been diagnosed with CKD, and the Cardiovascular Health Study showed that CKD was associated with a 37% higher rate of dementia after a six-year follow-up period [15, 16]. In the United States alone, more than 20 million people are affected with CKD and over 500,000 have end-stage renal disease [17].

Geroprotectors are compounds that slow the biological aging process and may therefore extend healthy lifespan [18]. Using the screening paradigm described above, we uncovered several potential geroprotectors, including the curcumin derivative J147 and the fisetin derivative CMS121 [19–23]. Both compounds elicit therapeutic cognitive effects such as memory enhancement in multiple rodent models of AD [22, 24–26], and J147 is currently in clinical trials for AD [27]. While the development of these compounds was done in the context of neurodegenerative diseases, true geroprotectors should positively affect multiple organ systems throughout the body. Initially we asked if J147 and CMS121 could promote lifespan extension in a model organism, *Drosophila melanogaster*. Next, we asked if the compounds could reduce old age-associated brain and kidney deficits in the senescence-accelerated SAMP8 mice, and if so, were the molecular pathways altered by drug treatments shared or distinct between the brain and kidney.

SAMP8 mice display an accelerated aging phenotype that serves as an excellent model for age-dependent disorders including neurological [28, 29], cardiac [30], hepatic [31], and renal [32] dysfunction. These mice develop early learning and memory deficits between 8-10 months of age and early-stage CKD by 12 months of age [28, 29, 32–34]. We have previously used this unique model to assay memory, brain aging, and dementia [22, 24, 26]. Importantly, because neurological dysfunction in these mice arises from accelerated aging and not from genetic manipulation of the canonical disease-associated proteins, SAMP8 mice may serve as a more appropriate model of sporadic AD/dementia than the commonly used transgenic models of familial AD.

Since AD-like pathology and early-stage CKD occur in SAMP8 mice, we examined the effects of J147 and CMS121 on the brain and kidney by feeding the compounds to the mice starting in the last quadrant of their lifespan, when they already have cognitive problems and are beginning to develop CKD. The goal of this project was to investigate the geroprotective effects of J147 and CMS121 in two different aging tissues. Furthermore, it was asked whether the levels of key proteins involved in aging demonstrated similar or distinct alterations across different tissues with age. It is shown here that J147 and CMS121 both improved physiological makers for brain and kidney health, but that the molecular pathways involved in these improvements were distinct between the two organs.

## RESULTS

### J147 and CMS121 promote lifespan extension in *Drosophila melanogaster*

**Figure 1:**
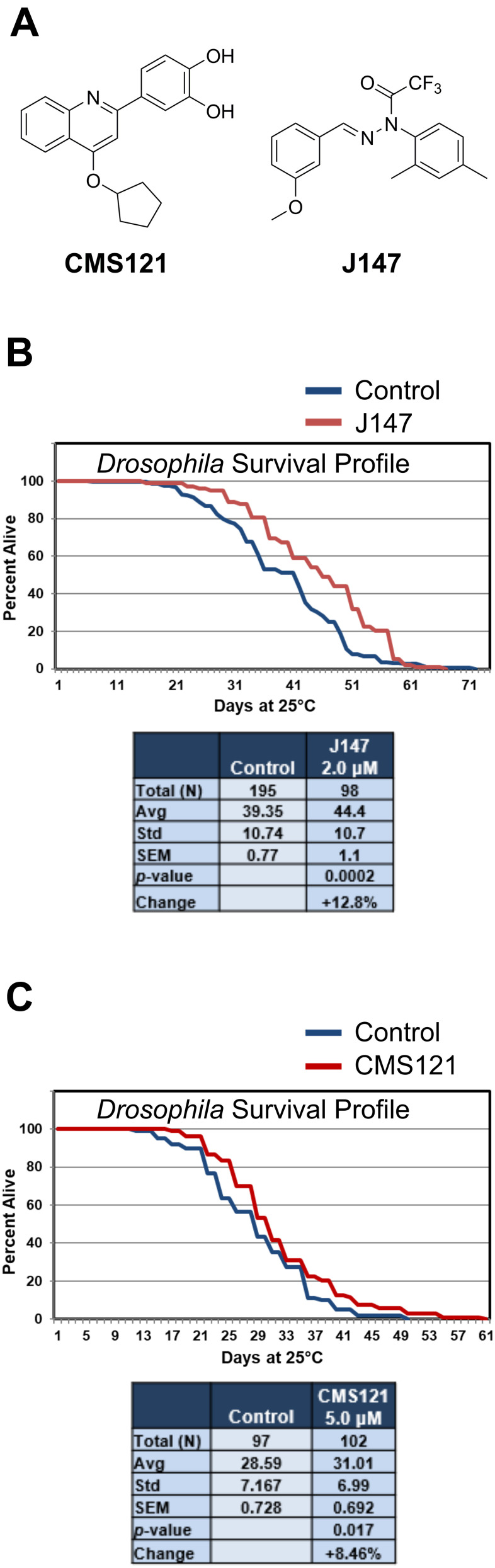
**J147 and CMS121 administration extend lifespan in D. *melanogaster*.** (**A**) Chemical structures of CMS121 and J147. (**B**) 2 μM J147 increased longevity in male *Drosophila* by 12.8% (*p* = 0.0002). The data in panel 1B is redrawn from Goldberg et al. [19]. (**C**) 5 μM CMS121 increased longevity in male *Drosophila* by 8.46% (*p* = 0.017). Results were compared by Student’s *t*-test.

J147 and CMS121 (Figure 1A) were selected because of their protective effects in a cell culture screening paradigm that mimics low energy metabolism, oxidative stress, proteotoxicity, reduced trophic factor support, and inflammation [8]: insults that increase with age. Thus, we asked if these compounds could promote lifespan extension. Due to its rapid life cycle and ease of maintenance in the laboratory, *D. melanogaster* is one of the most powerful model organisms for studying lifespan alterations [35]. Both J147 and CMS121 extended lifespan in *D. melanogaster* (Figure 1B, 1C). In the two independent studies, J147 (2 μM) elicited a 12.8% increase in survival (*p* = 0.0002) and CMS121 (5 μM) elicited an 8.46% increase in survival (*p* = 0.017). These data support the concept that both compounds have the potential to elicit beneficial effects on aging throughout an organism.

### J147 and CMS121 improve markers for both brain and kidney health

While geroprotectors extend lifespan, little is known about how these compounds affect different organ systems as they age. We utilized the rapidly aging SAMP8 mice to compare the effects of J147 and CMS121 on the brain and kidney. Female SAMP8 mice were divided into three treatment groups: those receiving control food, 200 ppm J147 food, or 400 ppm CMS121 food. The mice were placed on their respective diets at 9 months of age and were sacrificed at 13 months following behavioral assessment. For a comparison to younger controls, a cohort of 9-month-old mice were also sacrificed along with the three aged groups. Tissues from brain (cortex) and kidney were prepared for Western blot analysis. We then assessed the expression levels of 38 key proteins involved in aging and disease.

**Figure 2:**
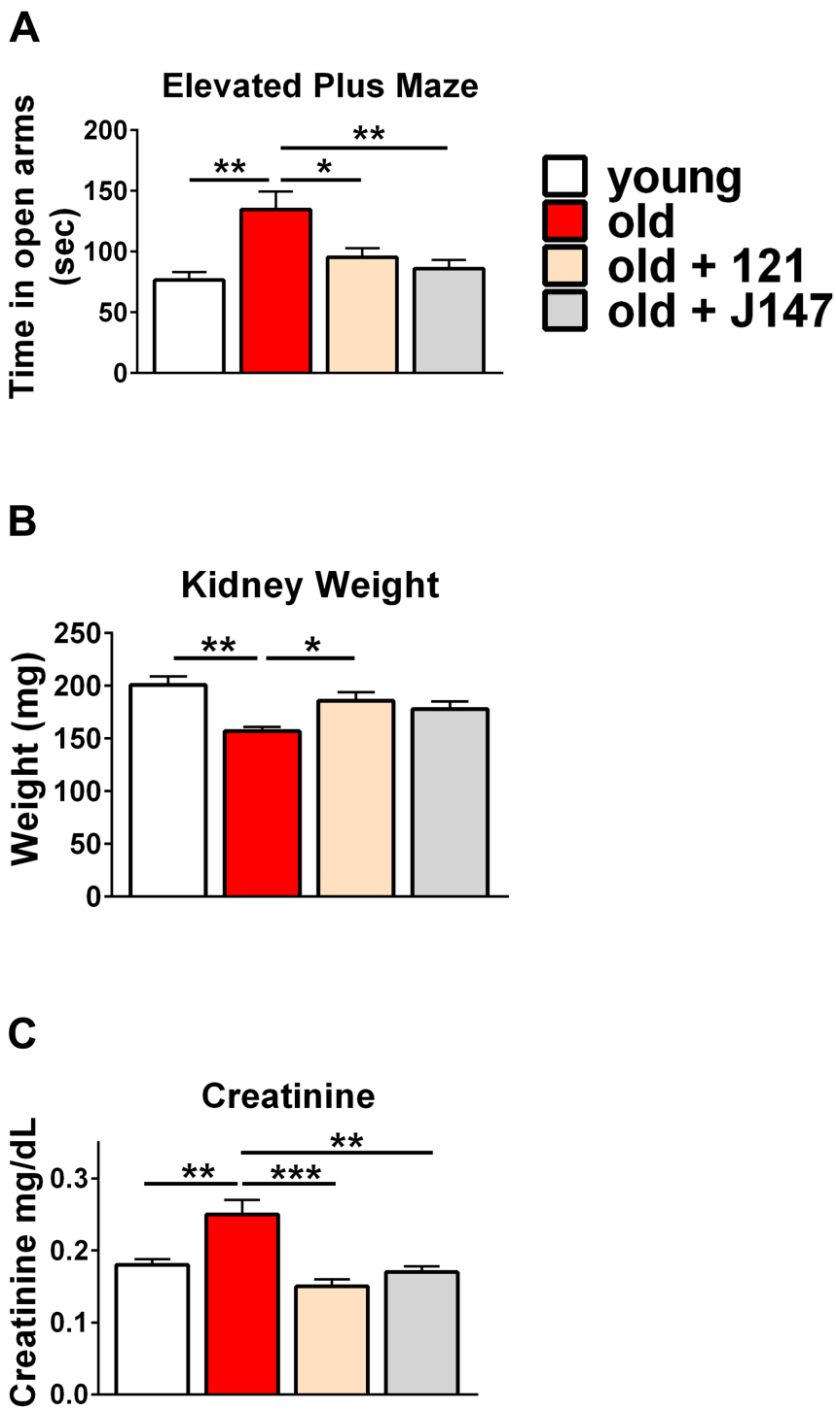
**J147 and CMS121 improve markers for brain and kidney health in old SAMP8 mice**. (**A**) Bar graph of time spent in the open arms of the elevated plus maze (n= 11-18/group). The data in panel 1A is redrawn from Currais et al. [26]. (**B**) Bar graph of measured kidney weights (n = 6/group). (**C**) Bar graph of measured plasma creatinine levels (n = 6/group). Data are presented as mean ± SEM. Results were compared by one-way ANOVA, followed by Turkey’s multiple comparison test.

To assess behavioral changes elicited by the compounds, we employed the elevated plus maze to measure the disinhibition behavior that frequently occurs in AD patients [36–38]. When untreated wild-type rodents are given the option to explore a plus maze, they tend to prefer spending time in the closed arms [39–41]. Closed arms give the animal a sense of security while the open arms evoke a state of vulnerability. Old mice spent significantly more time in the open arms compared to the young mice, mimicking the disinhibition behavior clinically observed in dementia [36–38]. Both compounds significantly reduced the time old mice spent exploring the open arms (Figure 2A).

The kidneys of SAMP8 mice experienced a mass decrease between 9 and 13 months of age. CMS121 treatment significantly restored kidney weight while J147 induced a similar, yet non-significant trend (Figure 2B). No significant differences were observed in body weight across the treatment groups throughout the study (Supplementary Figure 1). In addition, both drugs prevented an age-related increase in plasma creatinine levels, a strong clinical marker for beneficial therapeutic effects in kidney disease (Figure 2C).

### J147 and CMS121 elicit tissue-specific effects in the brain and kidney

**Figure 3:**
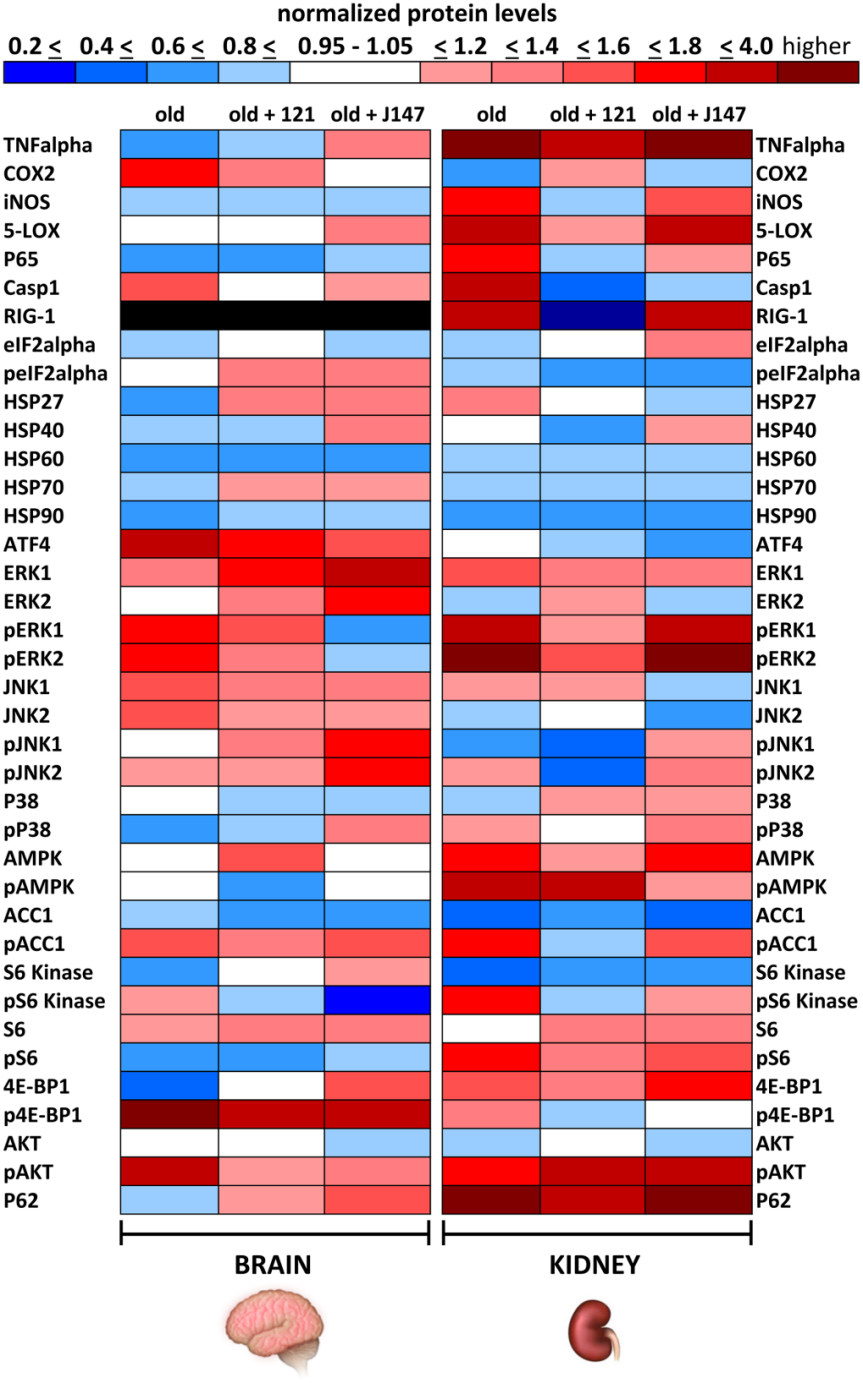
**J147 and CMS121 elicit tissue-specific effects in aged SAMP8 brain and kidney**. Heatmap of normalized protein levels in brain and kidney of old untreated and old compound-treated SAMP8 mice. Each non-phosphorylated protein was first normalized to beta-actin and then to its level in the same tissue of young mice. Phosphorylated proteins were first normalized to the total amount of the same non-phosphorylated protein and then to its level in young mice. Blue represents decreased protein levels, red represents increased protein levels, and white represents no change in protein level when compared to young mice. RIG-1 expression was not detectable in brain tissue (black). Mean data are presented (n = 4-6/group).

Because J147 and CMS121 improved markers for both brain and kidney health in old SAMP8 mice, it was next asked if they affected shared or distinct molecular pathways in the brain and kidney. To address this, Western blotting was used and the focus was on proteins from molecular pathways involved in aging and those that we have previously shown are affected by J147 in the hippocampus [22]. These included proteins involved in inflammation, proteostasis, and the MAPK and mTOR pathways. The proteins studied are listed in Figure 3 with the associated heatmap, and the Western blots of those with significant changes are discussed below. Western blots of all of the proteins are shown in Supplementary Figures 2, 3.

To better visualize protein level alterations induced by age and/or drug treatment across the brain and kidney tissues, we created a protein level heatmap. Each non-phosphorylated protein analyzed was first normalized to beta-actin and then to its level in young mice (as 1 Relative Level) to allow for a direct comparison of age-dependent protein level changes across the two tissues. Phosphorylated proteins were first normalized to the total amount of the same non-phosphorylated protein and then to its level in young mice. Deviations from 1 in the positive direction (increased abundance) are denoted by increasing shades of red, while deviations from 1 in the negative direction (decreased abundance) are denoted by increasing shades of blue. A five percent or less deviation from 1 in either direction is denoted by white, and could be considered as no change in the old animal relative to the young.

Extreme differences are represented by darker colors and moderate differences are represented by lighter colors. For example, in the aged brain, COX2 levels were increased by 70% relative to young mice and the band is colored red in the heatmap (Figure 3). In old mice treated with CMS121, COX2 brain levels were increased by only 36% relative to young mice and the band is colored dark pink. In old mice treated with J147, COX2 brain levels were increased by only 4% relative to young mice and the band is colored white. Because CMS121 was able to reduce the amount of COX2 found in old mice when compared to old untreated mice, its band is of lighter color than the old untreated band. However, because J147 was able to restore COX2 to young levels (less than 5% change), the band is white.

**Figure 4:**
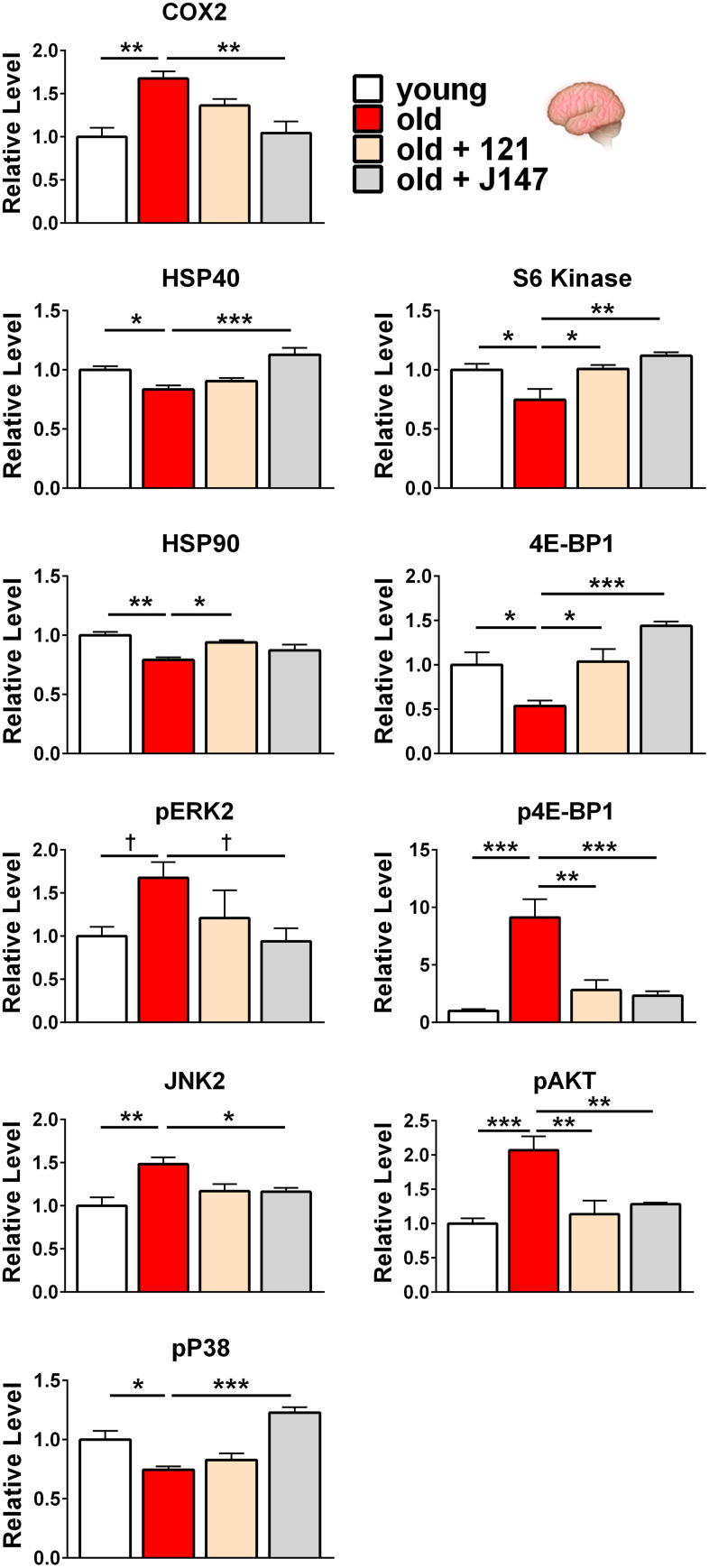
**J147 and CMS121 reverse age-associated protein level alterations in the brain involved in inflammation, proteostasis, and the MAPK and mTOR pathways**. Bar graphs of brain protein levels in young, old untreated, and old compound-treated SAMP8 mice. Data are presented as mean ± SEM (n = 4-5/group). Results were compared by one-way ANOVA, followed by Turkey’s multiple comparison test. Cross symbols represent significance when comparing 3 groups in ANOVA, excluding the unmarked treatment group from the analysis.

**Figure 5:**
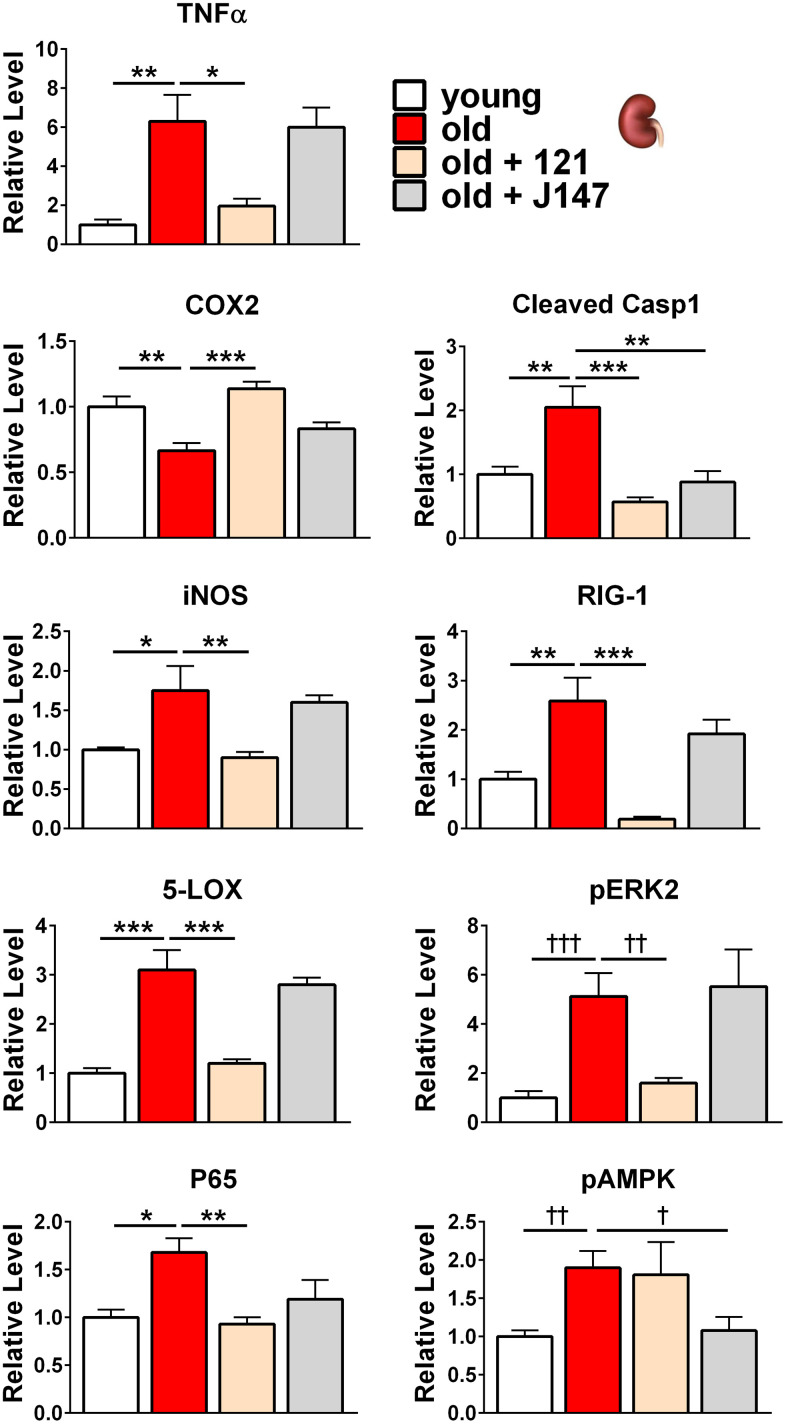
**J147 and CMS121 preferentially reverse age-associated protein level alterations in the kidney involved in inflammation, along with alterations to the MAPK and AMPK pathways**. Bar graphs of kidney protein levels in young, old untreated, and old compound-treated SAMP8 mice. Data are presented as mean ± SEM (n = 5-6/group). Results were compared by one-way ANOVA, followed by Turkey’s multiple comparison test. Cross symbols represent significance when comparing 3 groups in ANOVA, excluding the unmarked treatment group from the analysis.

We used a color-coding system that changes intensity for each 20% change in normalized protein level, up to a 2-fold change. However, this technique loses resolution for highly increased proteins such as phospho-4E-BP1 in the brain and TNFα in the kidney. Here, drug treatments substantially reduced protein levels in both instances (Figures 4, 5). However, because the drug treatment groups still had approximately 2-fold higher levels compared to young animals, these boxes are colored dark shades of red (Figure 3).

Aging induced tissue-specific protein level alterations in the SAMP8 brain and kidney, and alterations were generally more extreme in the kidney (Figure 3). Fifteen proteins showed decreased abundance in the aged brain (blue), fourteen proteins showed increased abundance (red), and eight proteins did not change with age (white). Within the aged kidney, thirteen proteins showed decreased abundance, twenty-two proteins showed increased abundance, and three proteins did not change with age. Sixteen of the proteins analyzed changed in the same direction with age across the two tissues, fifteen proteins changed in opposite directions or showed a substantial change in one tissue and no alteration in the other, and six proteins did not change with age across the two tissues. The P62 protein exhibited the largest age-dependent differential across the two tissues, with a 6% decrease in aged brain levels and an 11-fold increase in aged kidney levels. TNFα followed with a 40% decrease in aged brain levels and a 6-fold increase in aged kidney levels. On the other hand, some proteostasis-related proteins experienced very similar protein level alterations across the two tissues with age. HSP70, HSP90, and eIF2α exhibited a 10-25% decrease with age and each protein showed a 5% or less difference in level across the two aged tissues.

Compounds J147 and CMS121 elicited tissue-specific effects in the aged brain and kidney (Figure 3). Within the brain, the compound treatments reverted protein level alterations to a younger level in subcategories related to inflammation, proteostasis, and the MAPK and mTOR pathways (Figures 3, 4). Within the kidney, the compounds preferentially reversed inflammation-related protein level alterations, along with protein level alterations to the MAPK and AMPK pathways (Figures 3, 5). We will first describe the significant results related to these signaling pathways in the brain and then those in the kidney.

Regarding inflammation in the brain, COX2 levels were increased in old age and J147 reduced COX2 to levels comparable with young mice (Figures 3, 4). CMS121 elicited a trend towards decreased COX2 levels without reaching significance.

For proteostasis-related proteins, all heat shock proteins (HSPs) analyzed showed decreased levels in the old brain. In addition to their roles in stress responses, HSPs also monitor the cell’s proteins under non-stressful conditions by mediating proteolysis and helping newly synthesized proteins fold properly [42]. J147 increased HSP40 levels while CMS121 produced a non-significant increase. In contrast, CMS121 elevated HSP90 levels while J147 elicited a non-significant increase. Additionally, both compounds non-significantly increased both HSP27 and HSP70 levels (Figures 3, 4).

We next investigated the mitogen-activated protein kinase (MAPK) signaling cascade by assessing the levels of total and phosphorylated extracellular signal-regulated kinase (ERK1/2) in the brain cortex. Phosphorylated ERK2 was increased in the aged brain and J147 treatment decreased phosphorylation to young levels (Figure 3, 4). A similar trend was observed with phospho-ERK1, although significance was not reached when comparing young to old mice. CMS121 treatment elicited a non-significant trend towards decreased phospho-ERK1/2 levels.

We further explored the MAPK pathway in the brain by assessing levels of total and phosphorylated c-Jun N-terminal kinase (JNK1/2). JNK2 levels were increased in the old brain and J147 treatment restored more youthful levels (Figures 3, 4). CMS121 treatment followed the same pattern and nearly reached significance (*p* = 0.051). A similar, yet non-significant trend was observed for both compounds with JNK1 (Figure 3).

Next, we assessed levels of total and phosphorylated versions of the P38 protein in the brain. Phosphorylated P38 was decreased with age in the brain and levels were increased by J147, while CMS121 elicited a non-significant increase (Figures 3, 4).

Both compounds were able to normalize the levels of brain proteins altered with age within the mechanistic target of rapamycin (mTOR) pathway (Figures 3, 4). Total S6 kinase and 4E-BP1 levels were reduced in the old brain and the levels were significantly increased by both J147 and CMS121. Furthermore, phosphorylated 4E-BP1 was increased 9-fold in the aged brain and both J147 and CMS121 substantially reduced phosphorylation.

To continue our analysis of the mTOR pathway in the brain, we assessed the levels of total and phosphorylated AKT (also known as protein kinase B or PKB). Phosphorylated AKT was increased 2-fold in the aged brain and both J147 and CMS121 substantially reduced phosphorylation (Figures 3, 4).

In the SAMP8 kidney, all inflammation-related proteins analyzed showed significant changes in old age (Figures 3, 5). TNFα, iNOS, 5-LOX, P65, cleaved caspase 1 (CC1), and RIG-1 were all increased in the aged kidney and CMS121 treatment reverted the levels of all six proteins towards the young levels. J147 decreased CC1 levels and elicited non-significant decreases in TNFα, iNOS, 5-LOX, P65, and RIG-1 levels. Conversely, COX2 was decreased in the aged kidney. CMS121 increased COX2 to levels comparable with young mice while J147 elicited a non-significant increase.

Within the MAPK pathway, phosphorylated ERK2 levels were increased 5-fold in the old SAMP8 kidney. CMS121 treatment decreased phospho-ERK2 levels while J147 did not elicit an effect (Figures 3, 5). A similar, yet non-significant trend was observed for phospho-ERK1 (Figure 3).

We also assessed potential modulations to metabolism by analyzing total and phosphorylated AMP-activated protein kinase (AMPK) in the kidney. Phosphorylated AMPK levels were increased in the aged kidney and J147 treatment restored youthful levels while CMS121 did not elicit an effect (Figures 3, 5).

Together these data demonstrate that tissue-specific protein level changes occurred with age in the SAMP8 mice, and that these protein levels were modified by compounds that promote lifespan extension in *D. melanogaster*. The brain and kidney aged differently with many proteins moving in opposite directions across the two tissues with age, while J147 or CMS121 treatment altered protein levels in a distinct manner across the tissues that tended to restore the more youthful levels.

## DISCUSSION

The concept of geroprotectors posits that to reduce the rate of organismal aging, a geroprotector likely must reduce aging in multiple organ systems. To test this hypothesis, we first demonstrated the ability of J147 and CMS121 to extend lifespan in *D. melanogaster*. Next, we determined that the two compounds improved markers for both cognition and kidney function in old SAMP8 mice. Finally, we investigated a panel of key proteins involved in aging and disease to assess age-associated protein level alterations in the brain and kidney, and to determine if the molecular pathways of protection targeted by the compounds were shared between the tissues.

The brain and kidney heatmap show that the levels of many proteins moved in opposite directions across the two tissues with age alone (Figure 3). In fact, 15 of the 37 proteins analyzed in both tissues exhibited differential expression levels in old age, including TNFα, COX2, iNOS, 5-LOX, P65, HSP27, ATF4, JNK2, phospho-JNK1, phospho-P38, AMPK, phospho-AMPK, phospho-S6, 4E-BP1, and P62. Within this group of proteins, those in the brain generally showed decreased levels with age, while those in the kidney generally showed increased levels with age. However, COX2, ATF4, JNK2, and phospho-JNK1 were exceptions, with higher levels in the aged brain and lower levels in the aged kidney. The most extreme age-dependent changes in protein levels within this group of brain proteins included a 2-fold increase in ATF4, 70% increase in COX2, and a 47% decrease in 4E-BP1 levels in old animals. In the kidney, differences were more extreme and occurred with more proteins. P62 increased 11-fold, TNFα increased 6-fold, 5-LOX increased 3-fold, and iNOS, P65, AMPK, phospho-AMPK, and phospho-S6 levels increased 60 – 90% in the aged kidney.

These results are consistent with earlier studies showing that aging affects various tissues differently throughout the body. Shimoda et al. demonstrated that aging differentially altered the expression of angiogenic genes in a tissue-specific manner, with skeletal muscle and white adipose tissue experiencing the most pronounced alterations in aged mice [43]. Comparing various tissues in young and old mice, Hofmann et al. found that while Wnt signaling was generally decreased with age, a dichotomy existed in liver tissue with some aspects of Wnt-related signaling increased while others decreased with age [44]. Gosh and Thakur characterized receptor-interacting protein (RIP) expression in aged mice and found tissue-specific patterns. RIP expression decreased with age in the liver and kidney, increased with age in adipose tissue, and showed no age-dependent differences in the testis and prostate [45]. Using high-resolution isotope imaging, Arrojo E Drigo et al. demonstrated tissue- and cell-specific aging architectures in adult mice, characterized by a mosaic organization of young and old elements at the cell and protein level [46]. Thus, this study highlighted differences in biological age not only across tissues, but also between cells within a tissue and proteins within a cell.

More directly relevant to our data is a study that utilized high throughput RNA-sequencing technology to examine the changing transcriptome profiles of young and middle-aged *Drosophila* tissues [47]. The impact of both aging and intermittent fasting (IF) was assessed in neural and muscle tissues and transcriptional drift variance was analyzed to determine the progressive dysregulation of mRNA expression patterns. Principle component analysis of the neural and muscle mRNA found distinct non-overlapping transcriptome profiles between the two tissues and intermittent fasting resulted in a significant shift towards more youthful patterns and also reduced transcriptional drift variability when compared to non-fasted middle-aged controls. Age-related transcriptional changes in the neural tissues exhibited broader dysregulation of mRNA profiles, although the relative amplitude of changes was more pronounced in muscle tissues. Analysis of the dynamic expression differences revealed that there was minimal overlap between the neural and muscle tissues due to aging. Tissue-specific differences were also observed in the IF-dependent response, with neural tissues showing a greater shift towards more youthful expression patterns in middle-aged fasted cohorts, relative to those in the muscle. Further analysis revealed that accumulation of insoluble, ubiquitinated protein aggregates occurred at a greater extent in the neural tissues.

**Figure 6:**
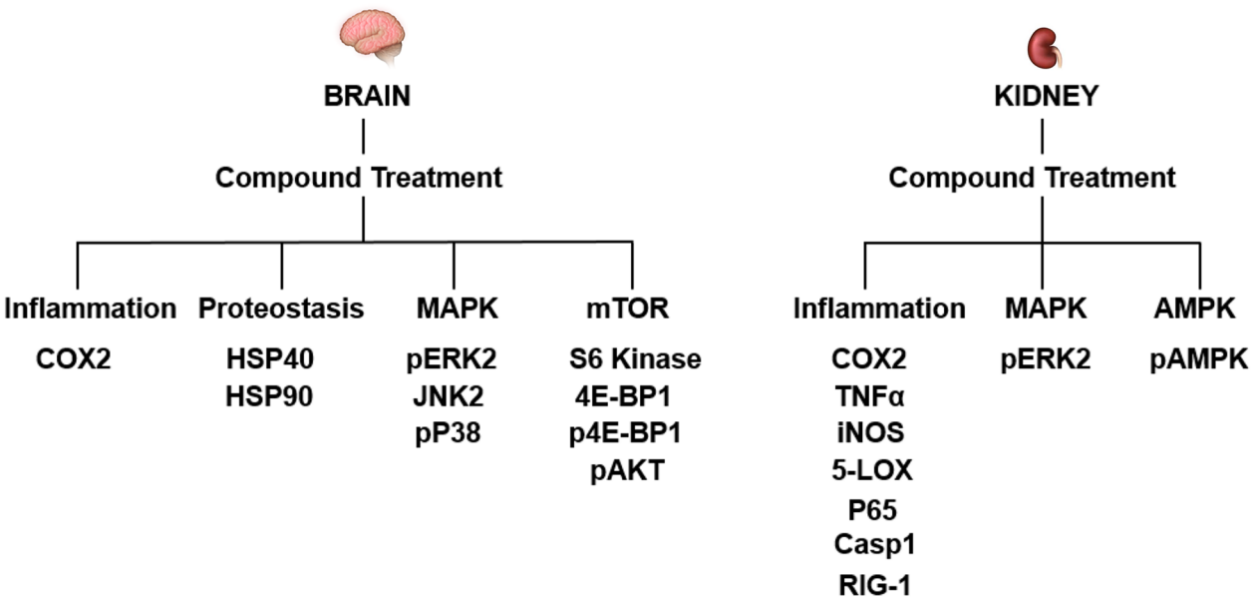
**The molecular pathways targeted by J147 and CMS121 are distinct between the brain and kidney.** Summary of proteins with significantly altered levels with age that were corrected by compound treatment. Proteins are listed under their respective subcategory.

Our study shows that while J147 and CMS121 treatments were able to prevent age-related phenotypes of organ dysfunction in the brain and kidney, they did so in a tissue-specific manner. Within the brain, compound treatment reversed protein level alterations to a younger level in subcategories related to inflammation, proteostasis, and the MAPK and mTOR pathways. However, the levels of inflammation-related proteins within the kidney were preferentially altered by compound treatment, along with alterations to the MAPK and AMPK pathways (Figure 6). Of the 38 proteins studied, 10 proteins showed significantly altered levels with age that were corrected by compound treatment in the brain and 9 proteins showed significantly altered levels with age that were corrected by compound treatment in the kidney. Aside from COX2 and phospho-ERK2, the remaining proteins targeted in each tissue were unique. Most importantly, instead of consistently increasing or decreasing the levels of a particular protein, J147 or CMS121 treatments tended to revert protein levels in the old mice to the levels of the young animal. For example, COX2 was increased in the aged brain and compound treatment reduced its level (Figure 4). Conversely, COX2 was decreased in the aged kidney and compound treatment increased its level (Figure 5). This nondirectional reversal effect shows that these compounds are differentially modulating molecular pathways in each tissue to restore homeostasis.

Diabetic kidney disease imposes structural changes to the kidney that results in altered kidney size, clinically observed as an initial hypertrophic phase [48, 49] that is followed by later-stage atrophy [50–52]. Additionally, structural and functional changes to the kidney are observed with normal aging and this can also lead to alterations in kidney size, seen as a decrease with age that is most apparent after the age of 50 [53–56]. Furthermore, atherosclerosis accelerates the decrease of renal size and increase of serum creatinine levels with age [57]. CKD, diabetic nephropathy, and ischemia-reperfusion injury are associated with tubular epithelial cell apoptosis-induced atrophy [51, 52, 58].

Inflammation has emerged as a major pathophysiological mechanism underlying diabetic kidney disease [59], thus it is likely that the anti-inflammatory effects of compound treatment on the kidney may have been the main mechanism of preserved kidney weight and function in the old mice (Figure 2B, 2C). It has been shown that renal hypertrophy in streptozotocin-induced diabetic rats [60, 61] and renal atrophy following ischemia-reperfusion injury in mice [58] could both be prevented by inhibiting TNFα signaling. Furthermore, studies with streptozotocin-induced diabetic rats treated with 1,25-dihydroxyvitamin D3 [62], cilostazol [63], or curcumin [64] demonstrated renoprotection via the blunting of NF-κB signaling. In our study, treatment with CMS121 reduced both TNFα and NF-κB P65 kidney levels, while J147 treatment elicited a non-significant decrease in both proteins (Figure 5). Additionally, a study of dogs with CKD reported decreased levels of COX2 in the diseased kidneys [65], in agreement with our SAMP8 data. We found that CMS121 treatment increased COX2 levels and J147 did so non-significantly. Non-steroidal anti-inflammatory drugs (NSAIDs) are well documented to induce nephrotoxicity when used chronically in elderly patients or those with comorbidities. This is due to COX inhibition, which inhibits the conversion of arachidonic acid into prostaglandins. Prostaglandins function as vasodilators in the kidney, increasing renal perfusion [66]. Thus, animals with an age-associated decrease in COX levels are likely experiencing inadequate renal blood flow, consequently impairing kidney function. Reports have also demonstrated protection to the kidney or cultured kidney cells by decreasing iNOS [67], 5-LOX [68], cleaved caspase 1 [69], or RIG-1 [70] levels, and all of these protein levels were reduced by compound treatment in our study (Figure 5).

Analysis of the MAPK signaling cascade showed that the compounds modulate phospho-ERK, JNK, and phospho-P38 protein levels in the aged brain and phospho-ERK protein levels in the aged kidney. Studies in rats have shown the MAPK pathway exhibits age-related impairments in both the brain [71] and kidney [72], and caloric restriction suppressed the age-related changes in both tissues. ERK is involved in a variety of crucial cellular and physiological processes including cell proliferation, differentiation, adhesion, migration, and survival. There are at least 270 substrates of ERK spread across all cellular compartments [73]. ERK is activated by dual phosphorylation followed by translocation to the nucleus where it then phosphorylates multiple transcription factors [74]. The JNK proteins function as critical regulators of transcription and are activated in response to environmental stress and growth factors. Additionally, JNKs play an important role in apoptosis [75]. Another member of the MAPK pathway, P38 is activated by inflammatory cytokines and plays an important role in the immune response. Additional activators include hormones, ligands for G protein-coupled receptors, osmotic shock, and heat shock [75].

Investigation of the mTOR pathway revealed that the compounds modulate S6 kinase, 4E-BP1, phospho-4E-BP1, and phospho-AKT protein levels in the aged brain. Age-related perturbations of the mTOR pathway in the brain affect multiple downstream pathways including glucose metabolism, energy production, mitochondrial function, cell growth, and autophagy: key players in age-related cognitive decline [76]. To stimulate protein synthesis, mTOR complex 1 phosphorylates S6 kinase and eukaryotic translation initiation factor 4E (eIF4E)-binding protein 1 (4E-BP1) [77]. mTOR complex 2 phosphorylates AKT to activate its regulation of diverse cellular processes. A variety of human diseases including neuronal degeneration, diabetes, cardiac hypertrophy, vascular disorders, and cancer are associated with dysregulation of AKT signaling, and thus a great effort has been directed toward development of ATK inhibitors [77, 78].

J147 modulated phospho-AMPK protein levels in the aged kidney. AMPK is able to monitor the available energy in a cell by directly binding adenine nucleotides. When AMPK is activated by a change in available energy, metabolism is redirected towards increased catabolism and decreased anabolism via phosphorylation of proteins in mTOR complex 1, lipid homeostasis, glycolysis, and mitochondrial homeostasis, in addition to targeting transcriptional regulators [79].

A common feature observed across the two tissues is a general age-related increase in transcription- and translation-related protein levels (Figure 3). This is most striking in the old brain, with an increased abundance of ATF4, ERK1, phospho-ERK1/2, JNK1/2, phospho-JNK2, and phospho-4E-BP1. Aside from this group, the majority of proteins in the brain experienced an age-related decrease. The kidney had an increased abundance of ERK1, phospho-ERK1/2, JNK1, phospho-JNK2, 4E-BP1, and phospho-4E-BP1; along with a decreased abundance of ERK2, JNK2, and phospho-JNK1. In many of these instances, treatment with J147 or CMS121 decreased the amount of total or phosphorylated protein levels. This leads us to speculate that dysregulations in transcription and translation may serve as primary age-related insults to the proteome that then have amplified downstream consequences. By modulating the levels of these proteins towards a more youthful level, J147 or CMS121 may elicit amplified protective effects. This notion is supported by multiple studies across diverse organisms demonstrating that directly lowering translation extends lifespan [80–86].

Limitations of this study include our lack of DNA analysis. Telomere length and epigenetic mechanisms such as methylation can serve as meaningful methods to quantify aging. However, we did recently report that SAMP8 mice treated with J147 or CMS121 exhibited increased acetylation of histone H3K9, a site linked to memory enhancement [26]. An additional limitation is that we only assessed female mice in this study. Because women disproportionately develop AD at higher rates than men, we chose to use female mice [87].

## CONCLUSIONS

This study demonstrates that two geroprotective AD drug candidates, J147 and CMS121, were able to promote a more youthful phenotype in both brain and kidney. These compounds were both discovered using the same phenotypic screening assays that mimic the toxic environment of old cells, and thus it is not unexpected that they share commonalities in their ability to promote cellular fitness and survival. However, both compounds appeared to function differently in the brain and kidney in order to maintain the more youthful phenotype, as defined by physiological markers and the fact that the compounds returned the levels of protein expression to those of younger mice. These data show that geroprotectors can promote the health of multiple tissues, but they may do so by employing different, tissue-specific molecular pathways. This is a valuable attribute, as many proteins demonstrated tissue-specific changes in their levels. Thus, compounds that elicit a unidirectional effect across all tissues may consequently ameliorate age-associated changes in one tissue while potentiating abnormalities in other tissues. Given the association between CKD and dementia [15, 16], therapeutics such as J147 or CMS121 that reduce age-associated decline in the kidney as well as the brain may be particularly clinically useful.

## MATERIALS AND METHODS

### Study design

The goal of this project was to investigate the geroprotective effects of J147 and CMS121 in two different aging tissues. Furthermore, it was asked whether the levels of key proteins involved in aging experienced similar or distinct alterations across different tissues with age.

We selected two structurally distinct compounds, J147 and CMS121, for this study because of their protective effects in cell culture assays mimicking age-associated toxicities [19–23]. Additionally, both compounds were previously shown to enhance cognition in multiple rodent models of AD [22, 24–26]. J147 and CMS121 are derivatives of the natural products curcumin and fisetin, respectively, with improved bioactivities. Because these are our laboratory’s top two AD drug candidates, we were particularly interested in assessing their effects in two distinct aging tissues in the SAMP8 mice.

The main risk factors for developing AD are age and gender [87]. Because women disproportionately develop AD at higher rates than men, we chose to use female mice in this study.

Mice were randomly assigned to experimental groups and the number of mice per group was determined based on previous experiments [22, 33] and was sufficient to attain statistical power. Twenty-three 9-month-old female SAMP8 mice received vehicle diet (LabDiet 5015, TestDiet, Richmond, IN), twenty-two 9-month-old female mice received CMS121 diet (LabDiet 5015 + 400 ppm CMS121, TestDiet), and twenty-two 9-month-old female mice received J147 diet (LabDiet 5015 + 200 ppm J147, TestDiet). Diet treatment lasted 4 months until the mice reached 13 months of age. SAMP8 mice present a strong phenotype at 9 months of age [22, 33, 88]. J147 was administered at 200 ppm (~10mg/kg/day), a dose previously effective in mouse models [20, 22, 25]. CMS121 was administered at 400 ppm (~20mg/kg/day), selected based on its greater efficacy compared to its parent, fisetin, in *in vitro* assays and positive results with 500 ppm fisetin in the SAMP8 mice [89]. The baseline control group consisted of eleven 9-month-old female SAMP8 mice. The effect of compound treatment was assessed in 13-month-old SAMP8 mice and age-related changes were defined by comparison to 9-month-old mice. Likely because the mice were near the end of their lifespan, fourteen mice died throughout the study, including 6 on control diet, and 4 on each of the compound-treated diets.

### SAMP8 mice

The SAMP8 line was acquired from Harlan Laboratories (U.K.). No significant differences were found between the groups when measuring mouse body weight regularly (Supplementary Figure 1). All experiments were performed in accordance with the US Public Health Service Guide for Care and Use of Animals and protocols were approved by the IACUC at the Salk Institute.

### Tissue preparation

Mice were anesthetized and blood was collected by cardiac puncture. Following perfusion with PBS, brains were removed and cerebral cortex was immediately dissected. Kidneys were removed and weighed. Cerebral cortex (referred to as brain) and kidney tissues were prepared for Western blotting.

### Western blotting

Western blots were performed as previously described [22]. All antibodies were from Cell Signaling except for ATF4 (Santa Cruz Biotechnology) and iNOS (BD Biosciences), and were used at a dilution of 1/1000 to 1/2000. The primary antibodies used were: 4E-BP1 (20 kD), phospho-4E-BP1 (20 kD), 5-LOX (78 kD), ACC1 (265 kD), phospho-ACC1 (265 kD), AKT (60 kD), phospho-AKT (60 kD), AMPK (62 kD), phospho-AMPK (62 kD), ATF4 (38 kD), cleaved caspase 1 (22 kD), COX2 (74 kD), eIF2α (38 kD), phospho-eIF2α (38 kD), ERK1/2 (44/42 kD), phospho-ERK1/2 (44/42 kD), HSP27 (27 kD), HSP40 (40 kD), HSP60 (60 kD), HSP70 (70 kD), HSP90 (90 kD), iNOS (130 kD), JNK1/2 (46/54 kD), phospho-JNK1/2 (46/54 kD), P38 (40 kD), phospho-P38 (40 kD), P62 (62 kD), NF-κB P65 (65 kD), RIG-1 (102 kD), S6 (32 kD), phospho-S6 (32 kD), S6 kinase (70 kD), phospho-S6 kinase (70 kD), and TNFα (17 kD). Horseradish peroxidase-conjugated secondary antibodies (goat anti-rabbit, goat anti-mouse, or rabbit anti-goat) (BioRad) diluted 1/5000) were used.

### Creatinine measurements

Plasma creatinine levels were measured by isotope dilution LC-MS/MS at the UAB-UCSD O’Brien Center for Acute Kidney Injury Research Bioanalytical Core.

### *Drosophila* care and lifespan analysis

F1 offspring from crosses between Canton-S and w^1118^ strains (w^1118/+^) were used in these studies. Male flies were collected and aged in same-sex cohorts (25 flies per vial) on standard media (agar, molasses, yeast, cornmeal, propionic acid, nipagin). Beginning at 1 week of age, flies were placed on standard fly media (control) or vials containing standard media with either 2 μM J147 or 5 μM CMS121. Flies were maintained at 25° C on a 12-hr:12-hr light:dark cycle for the study duration. Mortality data were used to generate Kaplan-Meier longevity curves.

### Statistical analysis

Data are presented as group mean ± standard error of the mean (SEM). Statistical analysis of the four groups was performed using one-way ANOVA followed by Turkey’s multiple comparison post-hoc test. For data regarding multiple time points, results were compared by two-way repeated measures ANOVA, followed by Bonferroni’s multiple comparison test. GraphPad Prism 6 was used and significance is indicated as *p < 0.05, **p < 0.01, and ***p < 0.001. Cross symbols represent significance when comparing 3 groups in ANOVA, excluding the unmarked treatment group from the analysis.

## Supplementary Material

Supplementary Figures
