# Supplementary material for: Geroprotective effects of Alzheimer’s disease drug candidates
Source: Aging (Albany NY). 2021 Feb 6;13(3):3269–89. doi: 10.18632/aging.202631 (PMC7906177; doi:10.18632/aging.202631)
Supplement: Supplementary Figures [file aging-13-202631-s001.pdf]

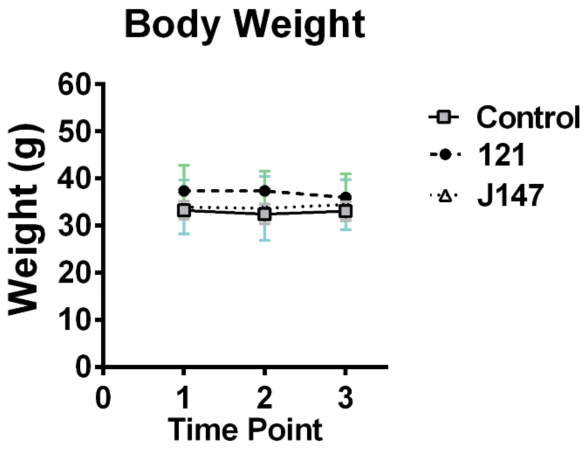

Supplementary Figure 1. Body weights of SAMP8 mice did not differ between treatment groups throughout the study. Nine-month-old female mice were fed with vehicle (n = 23), J147 (n = 22), or CMS121 (n = 22) diets until 13 months of age. Six mice receiving control diet, four mice receiving J147 diet, and four mice receiving CMS121 diet died throughout the study. No significant differences were found between the body weights of the groups at three different time points (9 months, 11 months, and 13 months). Data are presented as mean ± SD. Results were compared by two-way repeated measures ANOVA, followed by Bonferroni's multiple comparison test. This data is redrawn from Currais et al. [26].

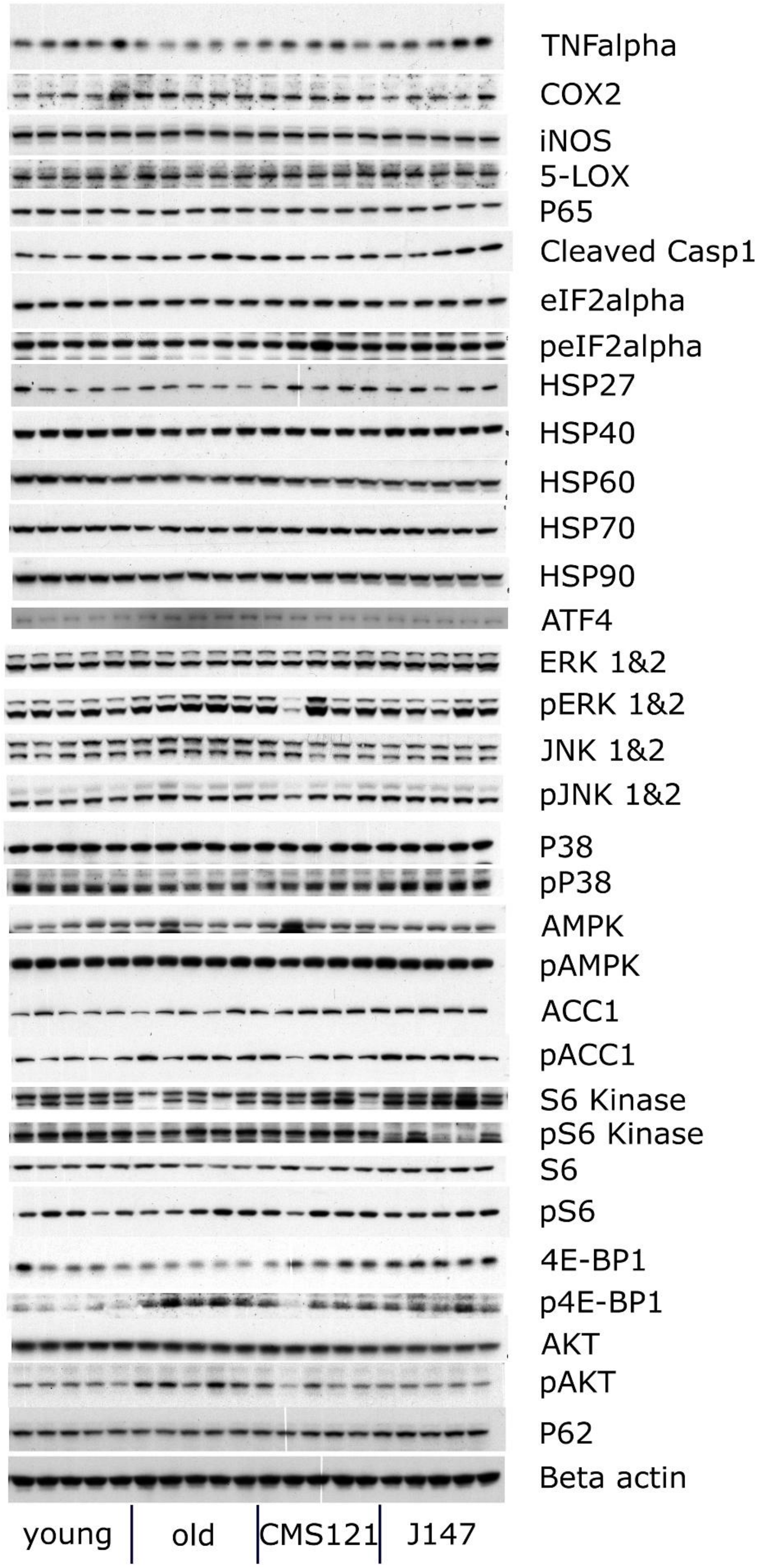

Supplementary Figure 2. J147 and CMS121 reverse age-associated protein level alterations in the brain involved in inflammation, proteostasis, and the MAPK and mTOR pathways. Raw Western blot data for brain proteins.

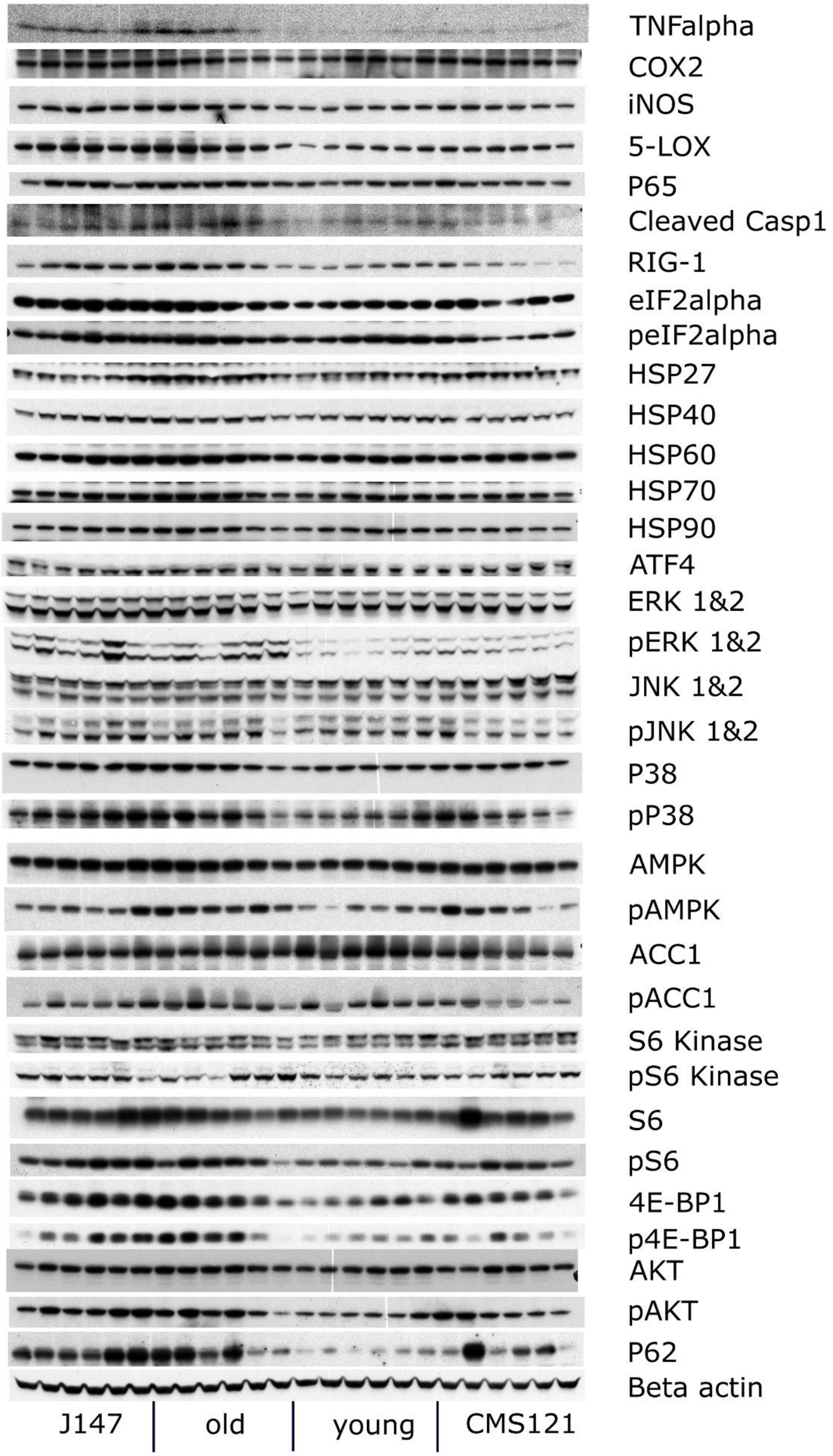

Supplementary Figure 3. J147 and CMS121 preferentially reverse age-associated protein level alterations in the kidney involved in inflammation, along with alterations to the MAPK and AMPK pathways. Raw Western blot data for kidney proteins.
